# Book Reviews

**Published:** 1898-04

**Authors:** 


### BOOK REVIEWS.

---

AMERICAN YEAR-BOOK OF MEDICINE AND SURGERY. Being a Yearly Digest of the Scientific Progress and Authoritative Opinion in all Branches of Medicine and Surgery, drawn from Journals, Monographs and Text-Books of the leading American and Foreign Authors and Investigators. Under the General Editorial Charge of George M. Gould, M.D. Published by W. B. Saunders, 925 Walnut street, Philadelphia. Price $6.50.

This work is well known to the profession, and therefore needs no introduction. The work certainly keeps abreast of the times, and is therefore very valuable in giving to the profession the advancements made in medicine and surgery during the year 1897. It embraces all departments of medicine, and is therefore a ready reference book for every physician. To add to the value of this work the collaborators are men of known ability and prominence. Such a book fills a long-felt want, as by it the physician can readily hunt up all the advances made during the year in any one particular subject, and therefore not be obliged to go through a whole mass of medical journals in order to obtain what he wishes. Besides, if he wishes to know more of the reference made, there is the reference also to the journal from which it comes and also the date. This work is unique, and no other journal takes its place. Every progressive physician should yearly add this book to his library.

---

DISEASES OF WOMEN. By Alexander J. C. Skene, M.D., LL.D., Professor of Gynecology Long Island Hospital Medical College. Third Edition. D. Appleton & Co , Publishers, New York. (Southern agency, Atlanta.)

To those in search of an extremely conservative text-book upon Gynecology this offers an excellent opportunity for securing such a book, but to those who wish to study the subject from the most advanced methods this is not the book for them to select. In the revision of this book it does seem that the author would have omitted the prolix details of illustrative cases which conclude so many of the chapters, as they consume space and could only be appreciated if they were seen and studied from a clinical standpoint.

Again, it seems a little strange to find in a text-boot on Diseases of Women a detailed description of Mr. B., whose bladder contained a foreign body, and at another place attention is called to one of the illustrative cases having his pulse accelerated to 106 degrees.

The illustrations and descriptions of plastic operations are excellent, and tend to show with a great deal of clearness just how these operations should be performed. There is an omission to a great extent of any very comprehensive description of the preparation of patients for operations, and the care necessary to secure cleanliness on the part of the operator and assistants.

The free use of opium in pelvic peritonitis and cellulitis is strongly advocated, although most gynecologists believe this to be a most pernicious practice.

This book, taken throughout, is not such a book as should be generally adopted as a text-book, but is one which will need a revision again within a very short period of time. E. C. D.

---

THE CARE AND FEEDING OF CHILDREN. By L. Emmett Holt, Professor of Diseases of Children, New York Polyclinic. D. Appleton & Co., Publishers, New York. (Southern agency, Atlanta.)

This little book, arranged in the form of a catechism, contains many very valuable and practical questions with the answers in a very concise style. This was first written for training-school for nursery maids, but its practical nature was early recognized by the members of the profession who have the care of children, and a demand has been made by them for it. It is a little book which those who treat many children would do well to read and to place within the reach of nurses who care for infants, especially during the early months of their lives. E. C. D.

THE YEAR-BOOK OF TREATMENT FOR 1898. A Critical Review for Practitioners of Medicine and Surgery. 488 pages. Cloth, $1.50. Lea Brothers & Co., Philadelphia and New York.

In this, the fourteenth annual issue of the "Year-Book of Treatment," the aim has been, as heretofore, to present the busy practitioner with a readable digest made of the progress in the domain of therapeutics during the past year. The entire domain of practical medicine is covered in a series of twenty-five chapters, each being assigned to a recognized authority, who gives all that is new, tried and true, with a critical statement of the comparative value and applicability of the various drugs, formulæ and methods of treatment. The work as a whole presents an exceedingly useful résumé of medical and surgical progress for the past year.

---

INTERNATIONAL MEDICAL ANNUAL. A Work of Reference for Medical Practitioners. Sixteenth year, 1898. E. B. Treat & Co., Publishers, 241 West 23d street, New York. [Price $3.00.]

In preparing the sixteenth annual issue of this work the editors and contributors have continued along the lines of previous editions, and they have presented a compact review of the progress of medical science (in 1897) as recorded in current medical literature. Those of our readers who are already familiar with this work will find this issue fully the equal of its predecessors. To our practitioners who wish to keep in touch with medical progress it is an exceedingly valuable book of reference.

---

MESSRS. LEA BROTHERS & CO., of Philadelphia, announce for early publication the following books by eminent authorities:

"A Manual of Otology." By Gorham Bacon, A.M., M.D., Professor of Otology in University Medical College, New York. With an Introductory Chapter by Clarence J. Blake, M.D., Professor of Otology in the Harvard Medical School, Boston, Mass.

"The Treatment of Surgical Patients before and after Operation." By Samuel M. Brickner, M.D., Visiting Surgeon of the Mt. Sinai Hospital, New York.

"The Principles of Treatment." By J. Mitchell Bruce, M.D., F.R.C.P., Physician and Lecturer on Materia Medica and Therapeutics at Charing-Cross Hospital, London.

"Diseases of the Nose, Throat, Naso-Pharynx and Trachea: A Manual for Students and Practitioners." By Cornelius G. Coakley, M.D., Professor of Laryngology in University Medical College, New York.

"Diseases of Women: A Manual of Non-Surgical Gynecology, designed especially for the use of Students and General Practitioners." By Francis H. Davenport, M.D., Instructor in Gynecology in the Medical Department of Harvard University, Boston.

"A Treatise on Gynecology." By E. C. Dudley, A.M., M.D., Professor of Gynecology in the Chicago Medical College, Chicago.

"A Text-Book of Anatomy." By American Authors. Edited by Frederick Henry Gerrish, M.D., Professor of Anatomy in the Medical School of Maine.

"Manual of Skin Diseases." By W. A. Hardaway, M.D., Professor of Skin Diseases in the Missouri Medical College.

"The Principles and Practice of Obstetrics." By American Authors. Edited by Charles Jewett, M.D., Professor of Obstetrics in the Long Island College Hospital, Brooklyn, N. Y.

---

No one who is interested in the best contemporary French literature can afford to miss the series of sketches and stories by Paul Bourget, which will begin in *The Living Age* for April 2. These sketches have been but recently published in France, and this is their first appearance in English dress. They are translated for *The Living Age* by William Marchant. They are extremely clever and characteristic.

---

A Thoughtful Answer.—"What's the first step toward the digestion of the food?" asked the teacher. Up went the hand of a black-haired little fellow, who exclaimed, with eagerness, "Bite it off!"—*American Kitchen.*

